# Dual functionalized hyaluronic acid micelles loading paclitaxel for the therapy of breast cancer

**DOI:** 10.3389/fbioe.2023.1230585

**Published:** 2023-08-03

**Authors:** Zhanbiao Liu, Xuejun Chen, Qian Jin, Min Li, Siqing Zhu, Yi Zhang, Defu Zhi, Yinan Zhao, Liqin Li, Shubiao Zhang

**Affiliations:** ^1^ Key Laboratory of Biotechnology and Bioresources Utilization of Ministry of Education, Dalian Minzu University, Dalian, China; ^2^ State Key Laboratory of NBC Protection for Civilian, Beijing, China

**Keywords:** hyaluronic acid micelles, paclitaxel, targeting, pH responsive, anti-breast tumor

## Abstract

Although many carriers for the delivery of chemotherapeutic drugs have been investigated, the disadvantages of passive targeting and uncontrolled drug release limit their utility. Herein, hyaluronic acid (HA) was hydrophobically modified to serve as a carrier for binding to cluster determinant 44 (CD44) overexpressed on tumor cell surfaces. Specifically, after deacetylation, HA was grafted to dodecylamine or tetradecylamine to afford amphiphilic zwitterionic polymer micelles, designated dHAD and dHAT, respectively, for the delivery of paclitaxel (PTX). The micelles were negatively charged at pH 7.4 and positively charged at pH 5.6, and this pH sensitivity facilitated PTX release under acidic conditions. The cell uptake efficiencies of the dHAD-PTX and dHAT-PTX micelles by MCF-7 cells after 4 h of incubation were 96.9% and 95.4%, respectively, and their affinities for CD44 were twice that of HA. Furthermore, the micelles markedly inhibited tumor growth both *in vitro* and *in vivo*, with IC_50_ values of 1.943 μg/mL for dHAD-PTX and 1.874 μg/mL for dHAT-PTX for MCF-7 cells; the tumor inhibition rate of dHAD-PTX (92.96%) was higher than that of dHAT-PTX (78.65%). Importantly, dHAD and dHAT micelles showed negligible systemic toxicity. Our findings suggest that these micelles are promising delivery vehicles for antitumor drugs.

## 1 Introduction

By the end of 2020, 7.8 million women had been diagnosed in the preceding 5 years, making breast cancer the most common cancer in the world. Globally, women lose more disability-adjusted life years to breast cancer than to any other type of cancer (Wild et al., 2020). Chemotherapy is among the most common treatments for malignancies. One frequently used chemotherapeutic drug is paclitaxel (PTX), a tricyclic diterpene alkaloid that inhibits cell homeostasis by increasing microtubule polymerization, which leads to cell division being blocked at the G2/M phase and subsequently to apoptosis (Abu Samaan et al., 2019). Nano formulated PTX is widely used for the treatment of breast, ovarian, lung, and cervical cancers, among others. Although PTX exhibits good activity against breast cancer cells, it also kills normal breast cells; in addition, it is neurotoxic, and the use of high doses can result in systemic toxicity and the development of drug resistance (Gornstein and Schwarz, 2014; Zhang et al., 2020).

To address these obstacles, researchers are currently focusing on preparing novel paclitaxel nano formulations such as polymer conjugates, micelles, emulsions, liposomes, and nanocrystals (Shao et al., 2015; Yang et al., 2017; Yousef et al., 2018; Large et al., 2021). Polymeric micelles are promising nanocarriers because they physically self-assemble in water. For example, amphiphilic copolymers can form core–shell micelles composed of a hydrophilic shell and a hydrophobic core, in which a hydrophobic drug can be encapsulated to prevent its efficacy from being affected by the external environment (Yin et al., 2015; Xu et al., 2016; Huo et al., 2020). Moreover, the hydrophilic shell can improve the water solubility of hydrophobic drugs, preventing the body’s defense system from clearing the drugs, thus prolonging the blood circulation time of the drugs, and such carrier can also improve the biocompatibility and bioavailability of hydrophobic drugs (Akhavan and Ghaderi, 2013). In addition, because of their nanoscale structures, micelles can accumulate at tumor sites by means of the enhanced permeability and retention (EPR) effect, thereby enhancing the antitumor effects of the encapsulated drugs (Maeda, 2017; Park et al., 2019; Li et al., 2021b; Du et al., 2021).

One polymer that is useful for chemotherapeutic applications is hyaluronic acid (HA), a naturally occurring glycosaminoglycan that is widely present in the human body. HA has a strong affinity for the cell-specific receptor protein CD44, which is overexpressed on the surface of cancer cells (Oh et al., 2010; Li et al., 2017; Li et al., 2021a; Ashrafizadeh et al., 2021). In addition, HA shows good biocompatibility and biodegradability and low immunogenicity (Dosio et al., 2016; Elamin et al., 2018; Choi et al., 2019). Therefore, it has been used as the basis for various tumor-targeted drug delivery systems. Nowadays, PTX delivery based on HA is mainly involves synthesizing HA targeted prodrugs and then conjugates with PTX (Zhong et al., 2016; Chen et al., 2018; Zou et al., 2022). This construction mode is complex and the drug system has a single function. However, the anti-breast cancer research on HA micellar self-assembly delivery of PTX has not been reported yet.

**FIGURE 1:**
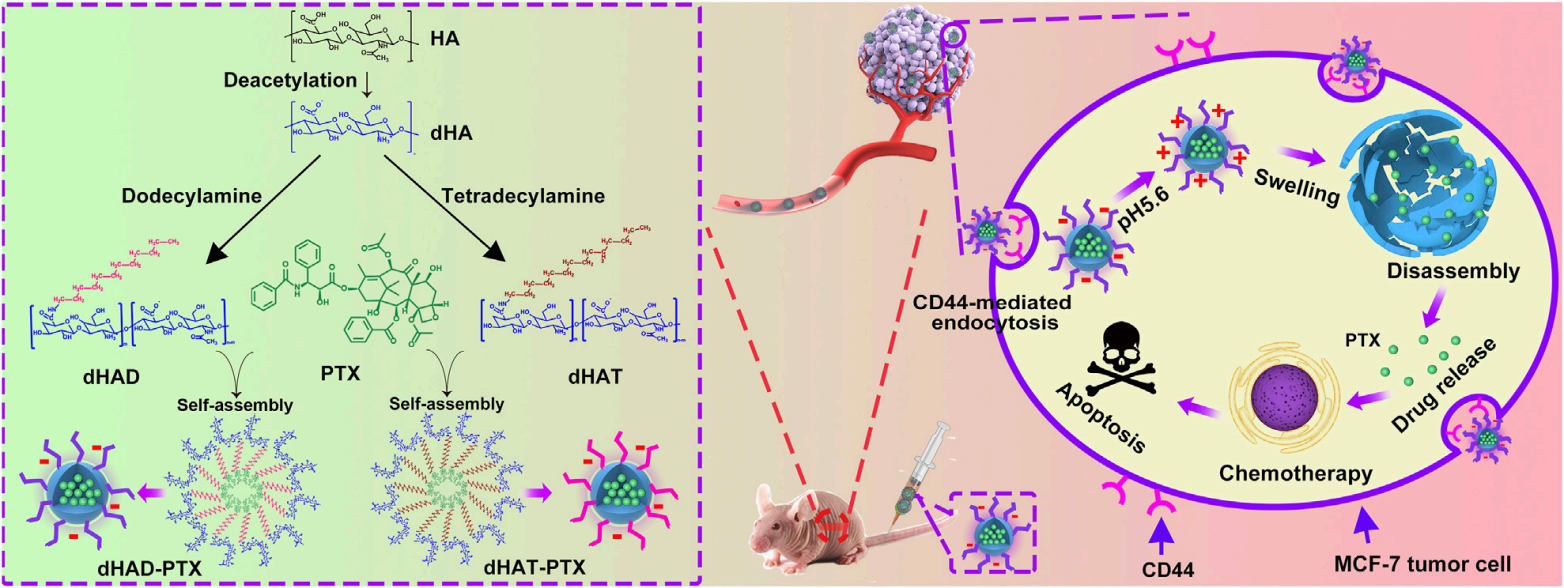
Preparation of dHAD-PTX and dHAT-PTX micelles, tumor targeting, and controlled intracellular release of PTX. dHAD and dHAT loaded with hydrophobic drug PTX in aqueous solution self-assembled into dHAD-PTX and dHAT-PTX micelles. Taking dHAD-PTX as an example to demonstrate the *in vivo* anti-tumor scheme of drug-loaded micelles. After administration, the dHAD-PTX accumulated at the tumor site by HA-mediated active targeting. After internalization, the dHAD-PTX responded to the acidic environment in the tumor cells, and PTX was released in a controlled manner.

In this study, alkyl amines were used to modify HA and two amphiphilic HA polymers (named dHAD and dHAT) were synthesized. Both amphiphilic polymers deliver PTX via self-assembly to form bifunctional micellar drugs in water (named dHAD-PTX and dHAT-PTX). On the one hand, micellar drugs can selectively target at tumor site through the tumor surface receptor CD44. On the other hand, the modified HA show potential reversal in tumor acid environment, causing micellar expansion and controlling the release of PTX from the micellar core (Huang et al., 2013) (Figure 1). The experiment confirmed that the alkylamine modified HA could improve the targeting of HA to CD44, and the uptake efficiency of tumor cells was significantly improved. Especially, dHAD-PTX and dHAT-PTX showed significant tumor inhibition in mice, with tumor inhibition rates reaching 92.96% and 78.65%, respectively. More importantly, the drug-loading micelles reduced the dose of PTX and significantly reduced toxicity. This work provides a new safe and effective way for the clinical application of HA-based PTX chemotherapy in the treatment of breast cancer.

## 2 Materials and methods

### 2.1 Materials

Hydrazine sulfate and PTX were purchased from Dalian Meilun Biotechnology Co. (Dalian, China). Sodium hyaluronate (MW 70 KDa) was obtained from Beijing Bloomage Biotechnology Co. (Beijing, China). Hydrazine hydrate, hydroiodic acid, iodic acid, dodecylamine, tetradecylamine, 4-(4,6-dimethoxytriazin-2-yl)-4-methylmorpholine hydrochloride, tetrahydrofuran, methanol, absolute ethanol, *N*,*N*-diisopropylethylamine, Tween80, and fluorescein isothiocyanate (FITC) were supplied by Shanghai Aladdin Biochemical Technology Co. (Shanghai, China). Cell counting kits (CCK-8) were provided by Beijing Solarbio Biotechnology Co. (Beijing, China). 4′,6-Diamidino-2-phenylindole (DAPI) was acquired from Merck & Co. Inc. (New Jersey, United States). Dulbecco’s Modified Eagle Medium (DMEM), fetal bovine serum (FBS), trypsin, and penicillin-streptomycin solution were obtained from Gibco (Grand Island, NY, United State). Recombinant human CD44 was purchased from Sino Biological Co. (Beijing, China). 1,1-Dioctadecyl-3,3,3,3-tetramethyl indotricarbocyanine iodide (DiR) was provided from Absin Bioscience Co. (Shanghai, China). The Protein Labeling Kit RED-NHS second Generation was purchased from NanoTemper Technologies (Beijing, China). The creatinine (CRE) kit, blood urea nitrogen (BUN) kit, aspartate aminotransferase (AST) kit, and alanine aminotransferase (ALT) kit were obtained from Biosino Bio-Technology and Science Co. (Beijing, China). Cell lines MCF-7 (human breast cancer cells) and HL7702 (human normal liver cells) were purchased from the Cell Bank of the Chinese Academy of Sciences (Shanghai, China).

### 2.2 Preparation of dHAD, dHAT, dHAD-PTX and dHAT-PTX micelles

Sodium hyaluronate (10 g) was allowed to react with hydrazine hydrate (300 mL) and hydrazine sulfate (5 g) under a nitrogen atmosphere at 70 °C for 72 h to obtain deacetylated HA (dHA). The carboxyl groups on dHA (400 mg) were activated by dissolving it in water (25 mL) with 4-(4,6-dimethoxytriazin-2-yl)-4-methylmorpholine hydrochloride (1.1 mmol) (D’Este et al., 2014), adding *N*,*N*-diisopropylethylamine (30 μL) dropwise to adjust the pH of the solution to 8, and stirring the reaction mixture in an ice–water bath for 3 h. After the reaction mixture warmed to room temperature, a solution of dodecylamine (1 mmol) or tetradecylamine (1 mmol) in tetrahydrofuran (25 mL) was slowly added dropwise to the activated dHA solution, and the reaction was allowed to proceed under nitrogen at room temperature for 30 h. The mixture was then poured into a dialysis bag (MWCO 12,000–14,000) and dialyzed in a 50% ethanol solution for 24 h, followed by a change of dialysate to deionized water for another 48 h. Subsequent lyophilization afforded the desired dHAD and dHAT polymers.

**FIGURE 2:**
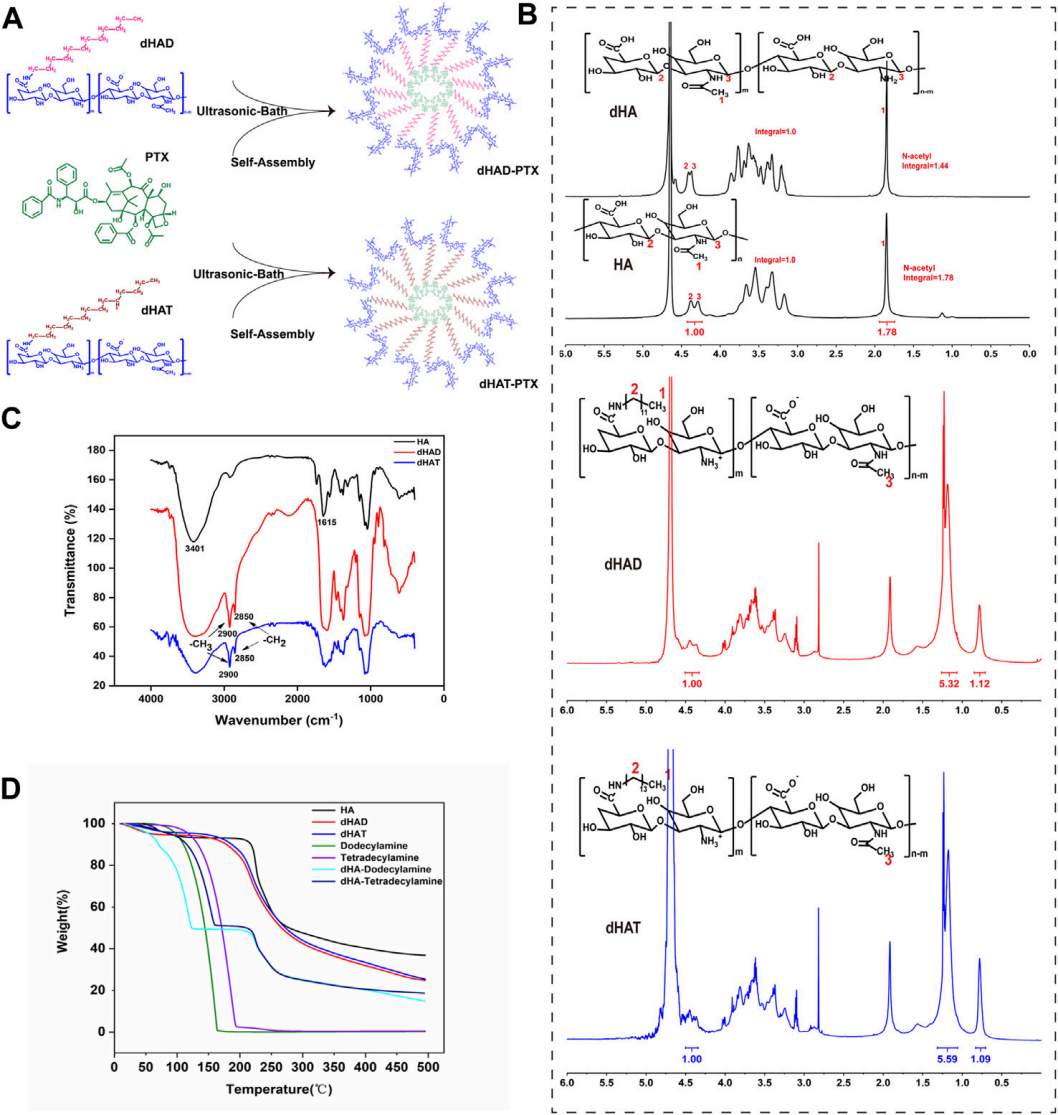
Synthesis and characterization of dHAD and dHAT. **(A)** Preparation process of dHAD-PTX and dHAT-PTX drug-loaded micelles. **(B)** ^1^H NMR spectra of HA and dHA (600 MHz, D_2_O). ^1^H NMR spectra of dHAD and dHAT (600 MHz, D_2_O). **(C)** FT-IR spectra and **(D)** thermogravimetric analysis of HA, dHAD, and dHAT.

PTX-loaded micelles were prepared by ultrasound and gradient dialysis (Figure 2A). Specifically, 10 mg of dHAT or dHAD was dissolved in enough ultrapure water to obtain a 2 mg/mL micelle solution. Then 1 mg of PTX was precisely weighed and dissolved in 5 mL of methanol, and the methanol solution was slowly added dropwise to the micelle solution. After sonication for 25 min in ultrasonic bath, the resulting solution was shaken for 4 h at 37 °C, transferred to a 7,000 Da dialysis bag, dialyzed with 40% methanol at room temperature for 24 h, dialyzed with deionized water for another 72 h, and then freeze-dried. The resulting dHAD-PTX and dHAT-PTX micelles were stored at 4 °C until use.

### 2.3 Characterization of dHAD, dHAT, dHAD-PTX and dHAT-PTX

The structures of the amphiphilic polymer dHA and the dHAD and dHAT micelles were characterized by Fourier transform (FT) IR (Thermo Scientific, Nicolet iN10, United States) and ^1^H NMR (600 MHz, D_2_O) (Bruker Avance III HD 600, Bruker, Swiss). In addition, the carriers were subjected to thermogravimetric analysis from 20°C to 500 °C under nitrogen at a heating rate of 10 °C/min.

The critical micelle concentrations (CMCs) of dHAD and dHAT were determined by means of pyrene fluorescence spectroscopy (Qiu et al., 2016). The particle size, zeta potentials and polymer dispersity index (PDI) of the dHAD, dHAT, dHAD-PTX and dHAT-PTX micelles were measured with a Zetasizer Nano ZS90 (Malvern Panalytical Co., Britain); each sample was analyzed three times. The morphologies of all the micelles were observed by transmission electron microscopy. The encapsulation efficiency (EE) and DL of the dHAD-PTX and dHAT-PTX micelles were investigated using a multimode microplate reader (Spark^®^, TECAN, Switzerland). The absorbances of the samples were measured at a wavelength of 227 nm, and the PTX concentrations in the micelles were calculated by substituting the absorbances into the standard curve for PTX. The following equations were used:

$$EE\left(\%\right)=\frac{Mass\;of\;PTX\;in\;micelles}{PTX\;dosage}\times 100$$ (1)

$$DL\left(\%\right)=\frac{Mass\;of\;PTX\;in\;micelles}{Total\;mass\;of\;PTX\;drug‐loaded\;micelles}\times 100$$ (2)

### 2.4 *In vitro* stability and hemolysis testing

To determine the serum stabilities of the dHAD and dHAT micelles, we sampled them daily for 7 days and determined their particle size by means of dynamic light scattering. Specifically, solutions of dHAD and dHAT micelles (1.0 mg/mL) were prepared in phosphate-buffered saline (PBS) at pH 7.4 and mixed with an equal volume of DMEM containing 10% FBS, and the mixtures were shaken for 2 h in a constant-temperature shaker at 37 °C and then stored at that temperature. In addition, the particle size of carriers stored at 4 °C were continuously measured to determine their stability upon storage at low temperature.

Hemolysis experiments on dHAD and dHAT were performed with fresh blood collected from female BALB/c nude mice. After the blood was collected, PBS was added, the blood was centrifuged at 3,200 rpm for 12 min, and the supernatant was discarded. This procedure was repeated five times, and then the pellet was resuspended in PBS to afford a red blood cell (RBC) suspension. The micelles and Tween 80 were separately diluted with the RBC suspension to generate solutions with concentrations of 0.1, 0.25, 0.5, 1.0, and 2.0 mg/mL, which were then incubated for 4 or 12 h at 37 °C. RBC suspended in PBS and ultrapure water, respectively, were used as negative control and positive control. After incubation, all the samples were centrifuged at 3,200 rpm for 12 min, and the absorbance of hemoglobin in the supernatant was measured at 541 nm with a multimode microplate reader. The hemolysis rate was calculated by means of the following equation:

$$Hemolysis\;rate\;\left(\%\right)=\frac{A_{t}-A_{n}}{A_{p}-A_{n}}\times 100\%$$ (3)

where *A*_*t*_, *A*_p_ and *A*_n_ are the absorbance of the sample, positive control (ultrapure water), and negative control (PBS).

### 2.5 pH responsiveness of dHAD-PTX and dHAT-PTX micelles

The pH responsiveness of the dHAD-PTX and dHAT-PTX micelles was assessed by changes in their particle size and zeta potentials in ultrapure water solutions at various pH values, which were prepared with 0.1 M HCl. dHAD-PTX and dHAT-PTX micelles solutions (1.0 mg/mL) were prepared by dissolving the micelles in ultrapure water at pHs ranging from 8.5 to 3.0. After the solutions were shaken in a constant-temperature shaker for 3 h, the particle size of the micelles was measured with a Zetasizer Nano ZS90. We also assessed the pH dependence of the zeta potentials of the micelles in the pH range from 8.5 to 3.0 at 25 C with a Zetasizer Nano ZS90; all samples were analyzed in triplicate.

### 2.6 Drug release from dHAD-PTX and dHAT-PTX micelles *in vitro*

The *in vitro* profiles of PTX release from the dHAD-PTX and dHAT-PTX micelles in pH 5.6 buffer (mimics tumor cell microenvironment) and in pH 7.4 buffer (mimics normal physiological environment) were assessed by means of dialysis (Johnson et al., 2013). dHAD-PTX and dHAT-PTX were dissolved in pH 5.6 and 7.4 PBS, respectively, and the concentration of the drug-loaded micelles was 0.1 M. The drug-loaded micelles (2.0 mL) were placed in a dialysis bag. Each dialysis bag was placed in a centrifuge tube containing 25 mL of phosphate buffer, and the tube was incubated in a constant-temperature shaker at 37 °C in the dark. At 0, 1, 2, 4, 6, 8, 10, 12, 16, 24, 36, 48, and 72 h, 100 µL of the release medium was removed from the external solution, and 100 µL of phosphate buffer at the corresponding pH was added to keep the total volume constant. The sample of release medium was transferred to a 96-well plate, and the absorbance of PTX was read with a multimode microplate reader and inputted into the standard curve to calculate the PTX concentration. The drug release rate was calculated according to the following equation:

$$E_{r}\left(\%\right)=\frac{V_{e}\sum_{1}^{n-1}C_{i}+V_{0}C_{n}}{m_{PTX}}\times 100$$ (4)

where *V*_*e*_ is the sample volume (0.1 mL), *V*_0_ is the initial volume (25 mL), *C*_*i*_ is the PTX concentration of the *i*th sample, *C*_*n*_ is the concentration of the *n*th sample, and *m*_PTX_ is the total amount of drug input (0.2 mg). To calculate the cumulative release of PTX, we prepared three samples for each of the two pH values.

### 2.7 Cell culture and tumor model establishment

Human breast cancer (MCF-7) and Human normal liver cells (HL7702) cells were cultured in DMEM high-glucose medium containing 10% FBS and 1% penicillin-streptomycin at 37 °C in a sterile incubator under 5% CO_2_. All of the animal experiments were approved by the Institutional Animal Care and Use Committee of the Research Institute of Chemical Defense (Beijing, China) (Approval No. LAE-2022-04-002). 5-Week-old female BALB/c nude mice purchased from the Jiangsu Gempharmatech Co. (Nanjing, China) were used to establish a tumor-bearing model by injecting MCF-7 cells into the axilla of the right forelimb of the mice (Holliday and Speirs, 2011).

### 2.8 *In vitro* cytotoxicity of dHAD, dHAT, dHAD-PTX and dHAT-PTX

A CCK-8 kit was used to evaluate the cytotoxicity of the empty and drug-loaded micelles to MCF-7 and HL7702 cells *in vitro*. MCF-7 cells and HL7702 cells were cultured under the same conditions. The cells were seeded into 96-well plates (5 × 10^3^ cells/well) and cultured in a cell incubator for 24 h. Then dHAD, dHAT, dHAD-PTX, or dHAT-PTX micelles diluted with fresh medium were added, and the cells were incubated for 48 h. After addition of the CCK-8 reagent and incubation for another 2 h, the absorbance at 450 nm was measured with a multimode microplate reader. Median inhibitory concentration (IC_50_) values were determined by using Prism 8.3.

### 2.9 Affinities of HA, dHAD and dHAT for CD44

The affinity of HA for CD44 was quantified by means of microscale thermophoresis (MST) (Jerabek-Willemsen et al., 2011). CD44 labeled with the Protein Labeling Kit RED-NHS second Generation was mixed with HA, dHAD, and dHAT at concentrations ranging from 33.4 to 0.00102 µM, respectively. After the mixtures were incubated for 1 h, 37 °C, they were loaded into Monolith NT.115 Capillaries (NanoTemper Technologies, Beijing, China), and dissociation constant was determined with MO.Control affinity analysis software (ver. 1.6). Then the MST measurement was performed using a Monolith NT.115 instrument (NanoTemper Technologies) at 20% LED power and high MST power. An MST-on time of 5 s was used for analysis. The CD44 concentration was 100 nM when the affinities for CD44 were assessed on the Monolith NT.115.1 × PBS (pH 7.4) and 0.05% Tween 20 constituted MST buffer. After detection of MST, the results were analyzed with MO.Affinity Analysis software (ver. 1.61).

### 2.10 Cellular uptake of dHAD-PTX and dHAT-PTX micelles

MCF-7 cells were digested by trypsin and then seeded into a confocal dish at a density of 3 × 10^4^ cells/well, and the dish was placed in an incubator for 24 h to bring the adherent cell density to at least 65%. Then FITC-dHAD-PTX and FITC-dHAT-PTX micelles (The method of FITC labeling micelles can be found in the Supplementary Material) were mixed with DMEM high-glucose medium at a PTX concentration of 0.01 μg/mL, and the mixtures were incubated for 0.5, 2, or 4 h. The medium was discarded, and the incubated cells was washed five times with 1× PBS. Cells were fixed with 4% polyformaldehyde and washed with 1× PBS three times. Cell nuclei were then stained with DAPI (100 μL) for 10 min in the dark, and the cells were washed four more times with 1× PBS. Micelle uptake was qualitatively analyzed by means of confocal laser scanning microscopy (CLSM; Leica, Wetzlar, Germany).

To quantify the cellular uptake of the dHAD-PTX and dHAT-PTX micelles, we seeded MCF-7 cells in a six-well plate at a cell density of 3 × 10^4^/well. The plates were incubated for 24 h to bring the adherent cell density to 80%. Drug-loaded micelles were added at the same PTX concentration used for the qualitative analysis described above, and the cells were incubated for 0.5, 2, or 4 h, washed with 1× PBS five times, and then digested with trypsin. The fluorescence intensities of the cells at various time points were quantified by flow cytometry (FCM; Becton-Dickinson, Heidelberg, Germany).

### 2.11 HA-mediated targeting study

MCF-7 cells were pretreated with HA (10 mg/mL) and cultured for 24 h. Pretreated cells were used as a control. The original culture medium was replaced with fresh medium containing FITC-dHAD-PTX or FITC-dHAT-PTX. After incubation for 4 h, the cellular uptake of micelles was qualitatively and quantitatively evaluated by CLSM and FCM (Li et al., 2022).

### 2.12 *In vivo* biodistribution of dHAD-DiR and dHAT-DiR micelles

BALB/c nude mice with MCF-7 tumors (∼80 mm^3^) were injected with saline, free DiR, dHAD-DiR, or dHAT-DiR (dHAD-DiR and dHAT-DiR were prepared by the method used to prepare dHAD-PTX and dHAT-PTX) via the tail vein at a DiR dose of 50 μg/kg. At a designated time after administration of the treatments, the mice were killed, and the tumors and the major tissues (heart, liver, spleen, lung, and kidney) were removed for imaging by means of an IVIS^®^ Spectrum *in vivo* imaging system (Waltham, United States).

### 2.13 *In vivo* antitumor effects of dHAD-PTX and dHAT-PTX micelles

We used BALB/c nude mice as animal models to investigate the antitumor effects of the dHAD-PTX and dHAT-PTX micelles *in vivo*. Nude mice (weight 18–20 g) were seeded with tumors by injection in the armpits, and tumor volume was monitored until it reached 100–120 mm^3^. Four experimental groups were established: a normal saline blank control group, a free-PTX group, a dHAD-PTX micelle group, and a dHAT-PTX micelle group (*n* = 6 for each group). PTX equivalent concentration doses of 5.0 mg/kg were administered intravenously to the different groups once every 4 days with 0.2 mL/20 g body weight during the 34 days treatment period (Figure 8A). Starting on the day of the first injection, the body weights and tumor volumes of the mice were measured and recorded every other day. The long and short diameters of the tumors were measured with Vernier calipers, and the tumor volumes were calculated to evaluate the antitumor effects of the dHAD-PTX and dHAT-PTX micelles by using the following equation:

$$V=\frac{W^{2}\times L}{2}$$ (5)

$$R=\frac{V_{C-}V_{n}}{V_{c}}\times 100\%$$ (6)

where *V* is the tumor volume, *W* is the tumor width, *L* is the tumor length, *R* is the tumor inhibition rate, *V*_C_ is the tumor volume of the control group, and *V*_*n*_ is tumor volume of the experimental group.

After 34 days of treatment, the mice were euthanized, and the major organs and the tumors were excised. The organ weights and the extents of tumor suppression were measured. Tumors and organs were fixed with 4% paraformaldehyde, embedded in paraffin, and sliced (slice thickness is 5 μm) by RM 2135 BioCut rotary microtome (Leica, Wetzlar, Germany), and the slices were stained with hematoxylin and eosin (H&E). Blood samples collected before the mice were euthanized were used for hematological tests: white blood cell (WBC), RBC, platelet (PLT), and hemoglobin (HGB) levels. Levels of serum ALT, BUN, AST, and CRE were also determined.

### 2.14 Statistical

Statistical analyses were carried out with Student’s t-test using GraphPad Prism 8.3 software. Data are presented as mean ± SD. Statistical significance values are as follows: *p < 0.05, **p < 0.01, ***p < 0.001, and ****p < 0.0001.

## 3 Results and discussion

### 3.1 Synthesis and characterization of dHAD and dHAT

The dHAD and dHAT micelles were synthesized as shown in Supplementary Figure S1. By controlling the feed ratio of the components and the reaction conditions, we could prepare carriers with the same HA deacetylation rates and similar grafting rates but different hydrophobic tails (Crescenzi et al., 2002; Gao et al., 2019). After HA was deacetylated, reaction with 4-(4,6-dimethoxytriazin-2-yl)-4-methylmorpholine hydrochloride (a condensing agent) and dodecylamine or tetradecylamine afforded pH-responsive amphiphilic HA polymers dHAD and dHAT. We maintained the deacetylation of HA at about 19% by controlling the feed ratio of hydrazine sulfate and HA, and then we synthesized dHAD and dHAT polymer carriers with grafting rates of 21%–22% according to a 1:1 feed ratio of dHA to dodecylamine or tetradecylamine.

Based on the integral ratio of the signal at 1.85 ppm (methyl protons) to that at 4.29–4.38 ppm (anomeric protons) in the ^1^H NMR spectra of HA and dHA (Figure 2B, top), the N-deacetylation degree of 19.1% can be obtained. (Zhang et al., 2013). The ^1^H NMR spectra of dHAD and dHAT showed new peaks at 0.85 and 1.23 ppm (Figure 2B, middle and bottom), which belong to the methyl and methylene peaks, respectively, of dodecylamine and tetradecylamine. Again, the ^1^H NMR data confirmed that the amines had been successfully grafted to dHA. The grafting ratios for dHAD and dHAT were determined from the ratios of the end-group proton peaks of dHA to the methyl peaks of dodecylamine grafted to dHAD and tetradecylamine grafted to dHAT; the ratios were 22.4% for dHAD and 21.8% for dHAT (Table 1).

**TABLE 1:** Properties of dHAD and dHAT polymers (mean ± SD, *n* = 5).

Polymer	DD^a^ (%)	GR^b^ (%)	CMC (mg/L)	Size (nm)	PDI	Zeta (mV)
dHAD	19.1	22.4	21.32	132.2 ± 1.6	0.103 ± 0.009	−25.8 ± 1.0
dHAT	19.1	21.8	11.72	173.1 ± 1.3	0.129 ± 0.007	−25.2 ± 1.3

Note.

^a^ *N*-deacetylation degree.

^b^ Grafting ratio.

In the FT-IR spectrum of HA, vibrational absorption peaks for the N–H on the N-acetylamino sugar ring and the O–H on the sugar ring appeared at 3,401 cm^−1^, and the C=O bending vibrational peak of the carboxyl group (–COOH) appeared at 1615^–1^ (Figure 2C). In contrast, the FT-IR spectra of dHAD and dHAT showed new characteristic peaks at 2900 and 2850 cm^−1^, which were attributed to the C–H vibrational absorption peaks for the–CH_3_ and–CH_2_ groups, respectively, of dodecylamine and tetradecylamine. The IR data confirmed that dodecylamine and tetradecylamine had been successfully grafted to the dHA.

Thermogravimetric analysis indicated that HA, dHAD, and dHAT showed an initial weight loss below 200 °C, which was due to the loss of physically bound water (Figure 2D). Subsequently, dHAD and dHAT showed substantial weight loss due to degradation at temperatures between 200°C and 500 °C. Furthermore, because dHAD and dHAT were grafted to dodecylamine and tetradecylamine, respectively, these polymers lost weight faster than HA but slower than physical mixtures of dHA with each amine (dHA-dodecylamine and dHA-tetradecylamine). The thermogravimetry results further confirmed that the amines were grafted to dHA.

### 3.2 Characterization of self-assembled dHAD, dHAT, dHAD-PTX and dHAT-PTX micelles

**FIGURE 3:**
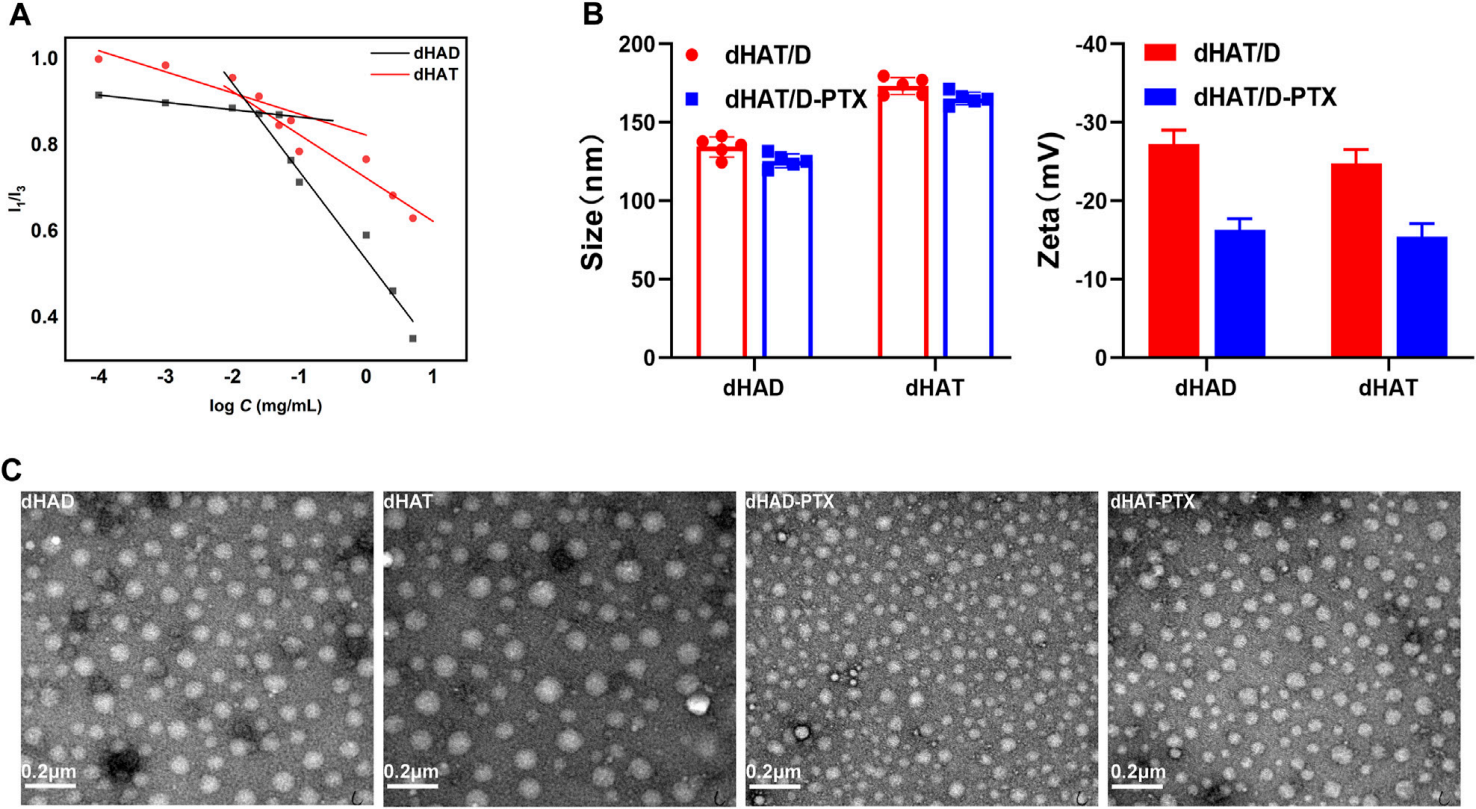
Characterization of dHAD, dHAT, dHAD-PTX, and dHAT-PTX micelles. **(A)** CMCs of dHAD and dHAT micelles. **(B)** Particle size and zeta potentials of dHAD, dHAT, dHAD-PTX, and dHAT-PTX micelles. **(C)** Transmission electron microscopy images of dHAD, dHAT, dHAD-PTX, and dHAT-PTX micelles.

As shown in Table 2, the DL of dHAD-PTX and dHAT-PTX were 13.56% and 13.45%, respectively. In aqueous solution, micelles can form only at concentrations above their CMC, which is therefore an important parameter for assessing whether amphiphiles can self-assemble into micelles. The CMCs of dHAD and dHAT were 21.32 mg/L and 11.72 mg/L, respectively, as measured by pyrene fluorescence spectroscopy (Figure 3A; Table 1). The CMC of dHAD and dHAT decreased significantly compared with that of ordinary surfactants, including that of poloxamer (1.0 × 10^3^–2.4 × 10^4^ mg/L) (Kratohvil et al., 1986).

**TABLE 2:** Properties of dHAD-PTX and dHAT-PTX micelles (mean ± SD, *n* = 5).

Micelle	EE^a^ (%)	DL^b^ (%)	Size (nm)	PDI	Zeta (mV)
dHAD-PTX	81.35	13.56	125.5 ± 1.0	0.129 ± 0.007	−16.6 ± 0.2
dHAT-PTX	80.72	13.45	165.3 ± 1.5	0.265 ± 0.005	−14.1 ± 0.4

Note.

^a^ Encapsulation rate.

^b^ Drug loading capacity.

The particle size of the dHAD and dHAT micelles that self-assembled in aqueous solution were 132.2 ± 1.6 nm and 173.1 ± 1.3 nm, respectively. Their PDIs were <0.3 (Figure 3B; Supplementary Figure S2A,B; Table 1), and their zeta potentials were −25.8 mV and −25.2 mV, respectively. Because of their negative charges, they repelled each other, which enhanced the stability of the system (Lee et al., 2008). The particle size of the dHAD-PTX and dHAT-PTX micelles were 125.5 ± 1.0 nm and 165.3 ± 1.5 nm, respectively, and their zeta potentials were −14.1 mV and −16.6 mV (Figure 3B; Supplementary Figure S2C,D; Table 2). Transmission electron microscopy indicated that all the micelles were spherical particles of uniform size (Figure 3C).

### 3.3 Stability and hemolysis of micelles

**FIGURE 4:**
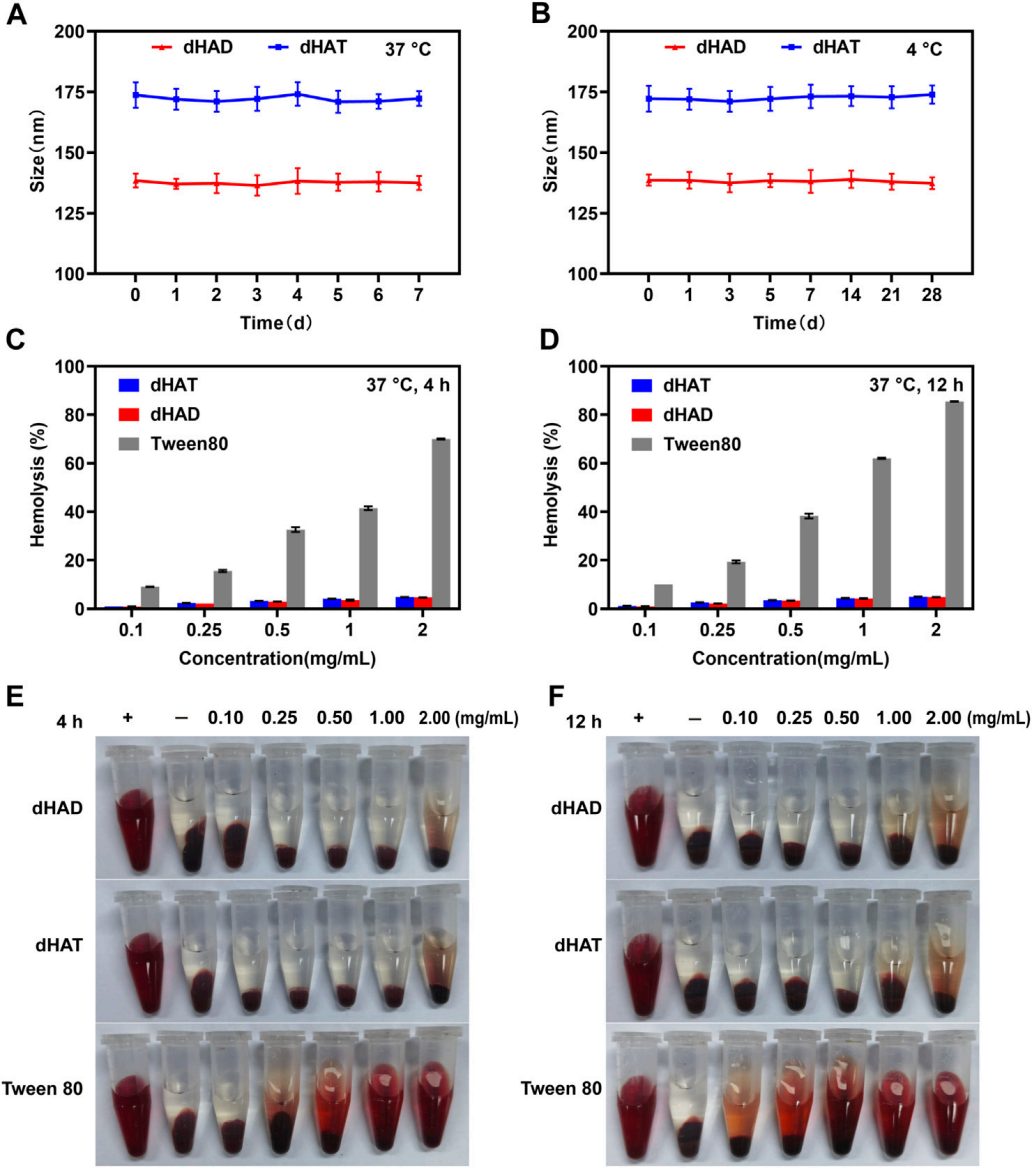
Assessment of the stabilities and hemolysis of the dHAT and dHAD micelles. **(A, B)** Changes in particles size of the micelles in DMEM containing 10% FBS over 7 days at 37 °C **(A)** and in PBS over 28 days at 4 °C **(B)**. **(C and D)** Concentration dependence of hemolysis rates of dHAT, dHAD, and Tween 80 micelles after 4 h **(C)** and 12 h **(D)**. **(E, F)** Pictures of dHAD and dHAT micellar hemolysis after 4 h **(E)** and 12 h **(F)**.

Micelles must be stable in blood to avoid *in vivo* toxic side effects and aggregation by interaction with fibrin, which can trigger vascular obstruction and other complications (Choi et al., 2010; Wang et al., 2013; Ghosh et al., 2014). We assessed the serum stabilities of the dHAD and dHAT micelles by monitoring changes in their particle size upon incubation in FBS-containing DMEM (without phenol red) for 7 days at 37 °C, and the storage stabilities were also assessed by monitoring the particle size changes over the course of storage for 28 days at 4 °C (Figures 4A,B). Upon incubation of the dHAD and dHAT micelles at 37 °C, their particle size remained basically unchanged, indicating they had good serum stability, did not aggregate, and could be expected to safely deliver PTX to tumor cells. The particle size also remained basically unchanged after 28 days of storage at 4 °C in pH 7.4 PBS, indicating that the micelles remained stable during storage at this temperature.

We assessed the safety of the micelles by investigating their hemolysis rates (Figures 4C–F). Micelles that are not biocompatible can damage the erythrocyte membrane, which leads to hemolysis and hemoglobin release. Tween 80 is often used as a control material to evaluate the hemolytic properties of carrier materials (Seo and Kim, 2010; Lopresti et al., 2011). We found that after Tween 80 was incubated for 4 h with fresh blood from BALB/c nude mice, its hemolysis rate at concentrations between 0.1 and 2 mg/mL ranged from 9.04% to 69.97%; and after incubation for 12 h, the rate ranged from 10.1% to 85.5%. In contrast, the hemolysis rates of both dHAD and dHAT were less than 5% after 4 or 12 h of incubation, indicating that the micelles had excellent biocompatibility.

### 3.4 pH sensitivities and *in vitro* drug release behavior of dHAD-PTX and dHAT-PTX micelles

**FIGURE 5:**
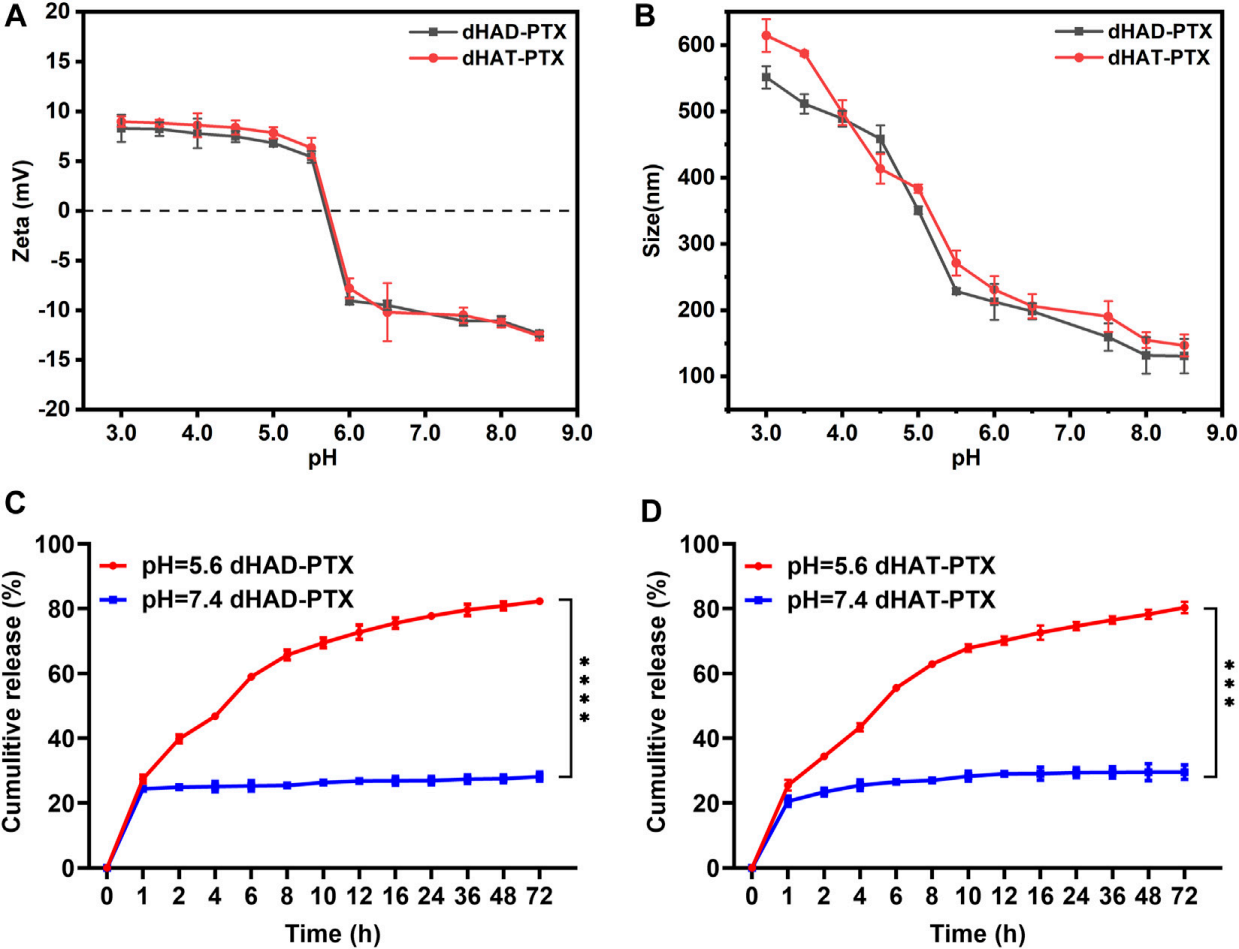
**(A, B)** Effects of pH on zeta potentials **(A)** and particle size **(B)** of dHAD-PTX and dHAT-PTX micelles at 37 °C. **(C, D)** Curves showing PTX release from dHAD-PTX micelles **(C)** and dHAT-PTX micelles **(D)** in PBS buffer at pH 7.4 and 5.6 (mean ± SD, n = 3; **p < 0.01, ***p < 0.001, ****p < 0.0001).

We assessed the pH sensitivities of the dHAD-PTX and dHAT-PTX micelles by measuring their zeta potentials and particle size at various pH values. The free amino groups on the micelle surfaces gradually became protonated when the pH dropped to 5.4–5.6, which in turn led to changes in the micelle surface charges: from −12.37 mV to 8.37 mV for the dHAD-PTX micelles and from −12.60 mV to 8.98 mV for the dHAT-PTX micelles (Figure 5A; Supplementary Figure S4A,B) (Huang et al., 2013). As shown in Figure 5B, Supplementary Figure S3A,B and Supplementary Table S1, the particle size of the dHAD-PTX and dHAT-PTX micelles increased with decreasing pH, from 130.8 nm to 551.6 nm and from 147.0 nm to 614.6 nm, respectively, owing to protonation of the free amino groups and the resulting increases in positive charge and electrostatic repulsion, which led to swelling and even dissociation of the micelles (Johnson et al., 2013).

The release of PTX from the dHAD-PTX and dHAT-PTX micelles was investigated in PBS buffer at pH 7.4 (normal physiological pH) and 5.6 (pH in tumor cell lysosomes). As shown in Figures 5C,D, the release rates of PTX were significantly different at pH 7.4 and 5.6. At pH 7.4, the PTX in the drug-loaded micelles was released slowly after 24 h, but the cumulative release amount was less than 30%, indicating that the micelles could be expected to remain stable before reaching the tumor site. In contrast, at pH 5.6, the amino groups became protonated, which resulted in swelling of the micelles and facilitated PTX diffusion into the medium. At pH 5.6, PTX release from the dHAD-PTX micelles (82.3%) was slightly faster than PTX release from the dHAT-PTX micelles (80.3%). This difference may have been due to the difference in their particle size resulting from their different hydrophobic tails. The results confirmed that the dHAD-PTX and dHAT-PTX micelles were pH responsive and could promote the release of the encapsulated PTX in tumor cells, thereby enhancing effect of the drug.

### 3.5 *In vitro* cytotoxicity of dHAD, dHAT, dHAD-PTX and dHAT-PTX

**FIGURE 6:**
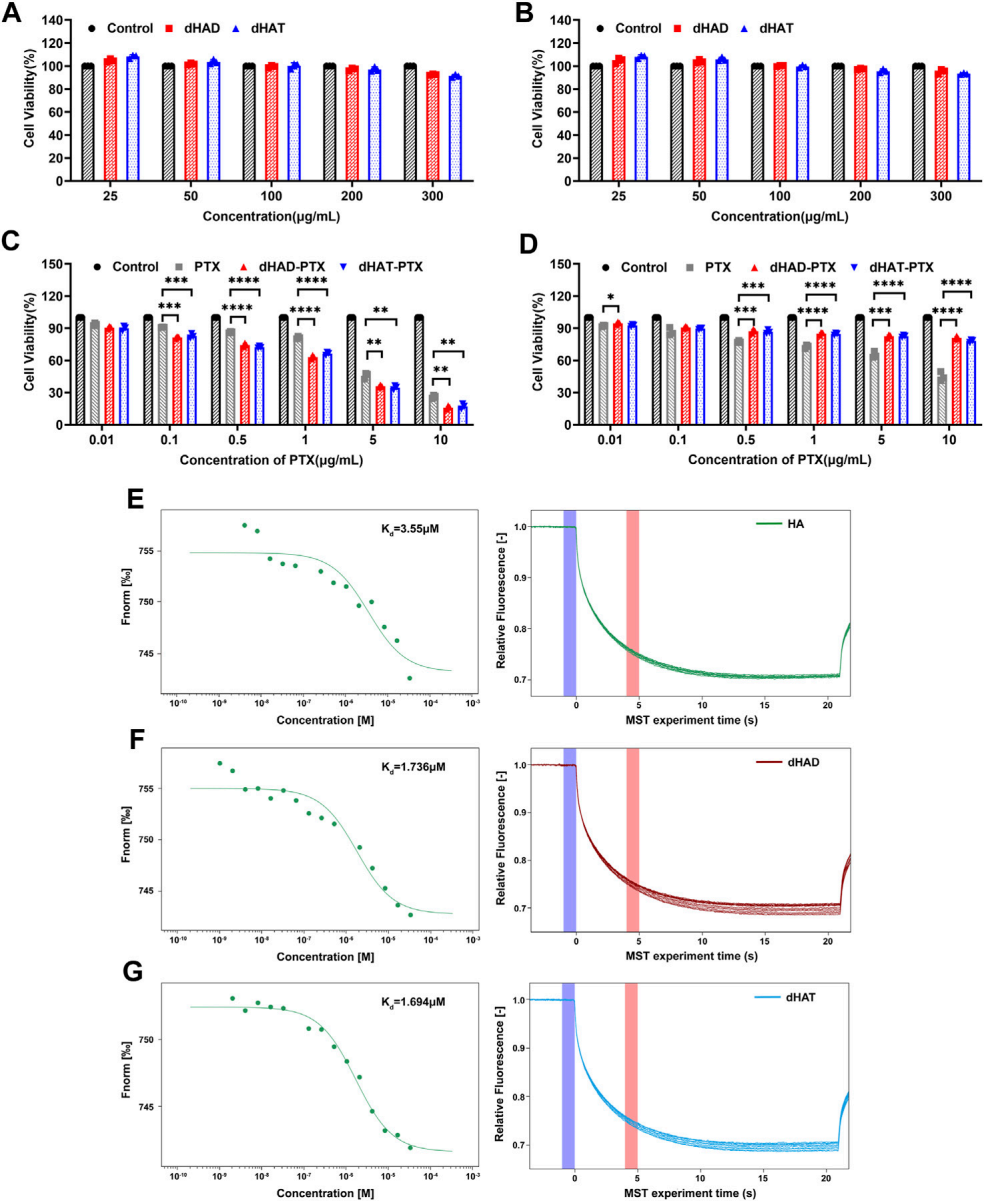
*In vitro* cytotoxicity of micelles to MCF-7 and HL7702 cells and MST assays of binding affinities of HA, dHAD, and dHAT for CD44. **(A, B)** *In vitro* cytotoxicity of dHAD and dHAT micelles to MCF-7 cells **(A)** and HL7702 cells **(B)**, as determined by the CCK-8 method. **(C, D)** Inhibition of proliferation of MCF-7 cells **(C)** and HL7702 cells **(D)** by free PTX and by dHAD-PTX and dHAT-PTX micelles, as determined by the CCK-8 method. Values in panels A–D are mean ± SD (n = 3; *p < 0.05, **p < 0.01, ***p < 0.001 and ****p < 0.0001 relative to the free paclitaxel treated group). **(E–G)** Equilibrium constant (K_d_) values for binding of HA **(E)**, dHAD **(F)**, and dHAT **(G)** to CD44.

We assessed the cytotoxicity of the dHAD and dHAT micelles against two cell lines (HL7702 and MCF-7) by means of the CCK-8 method. After incubation cells with dHAD or dHAT micelles at concentrations of 25–300 μg/mL for 48 h (Figures 6A,B), the viabilities of the cells treated with the two types of micelles exceeded 93% (HL7702) and 91% (MCF-7), respectively, confirming their safety and applicability as drug carriers.

Inhibition of the growth of MCF-7 and HL7702 cells by free PTX and by dHAD-PTX and dHAT-PTX micelles was evaluated after treatment for 48 h (Figures 6C,D). Free PTX showed a dose-dependent inhibitory effect on both cell lines. In contrast, although the dHAD-PTX and dHAT-PTX micelles exhibited significant inhibitory effects against the tumor cells (MCF-7), the viability of normal cells (HL7702) exceeded 90%. These results suggest that the dHAD-PTX and dHAT-PTX micelles can selectively kill tumor cells via interaction with HA receptors, making the toxicity of the micelles to normal cells negligible, a possibility that was confirmed by the results described in experiment of *in vivo* evaluation of dHAD-PTX and dHAT-PTX micelle toxicity. The IC_50_ values for free PTX and the dHAD-PTX and dHAT-PTX micelles against MCF-7 cells were 3.256 ± 0.131, 1.943 ± 0.231, and 1.874 ± 0.146 μg/mL, respectively. The micelles with the same PTX equivalent dose had stronger inhibitory effects than free PTX (Supplementary Figure S5) because they allowed for microenvironment-responsive release of PTX. More importantly, the dHAD-PTX micelles showed greater efficacy than the dHAT-PTX micelles (Supplementary Figure S6). This difference may have been due to the smaller particle size and higher drug-loading capacity of the former (Supplementary Figure S7; Tables 1 and 2), and the more sensitive pH response of the former micelles may have allowed them to release PTX more effectively.

### 3.6 Affinities of HA, dHAD and dHAT for CD44

To assess the affinities of HA, dHAD, and dHAT for CD44, we conducted an MST experiment in which the concentration of RED-labeled CD44 was kept constant at 100 nM, and the concentration of the unlabeled binding partner (HA, dHAD, or dHAT) was varied between 33.4 and 0.00102 µM. On the basis of the results of these experiments, the *K*_d_ values derived for the interactions of CD44 with HA, dHAD, and dHAT were 3.550 μM, 1.736 μM, and 1.694 μM, respectively (Figures 6E–G). A desirable feature of HA-CD44 binding has been reported to be the ubiquitous presence of a hydrophobic core (Banerji et al., 2007). In our work, we increased the hydrophobicity of HA by grafting dodecylamine and tetradecylamine. Thus, dHAD and dHAT were stronger targeted to CD44 compared with HA, which is consistent with reported studies (Bhattacharya et al., 2017). Then, the affinity between dHAD and dHAT with CD44 protein was higher than that between HA and CD44 protein and about twice that of HA.

### 3.7 Cellular uptake of dHAD-PTX and dHAT-PTX micelles

**FIGURE 7:**
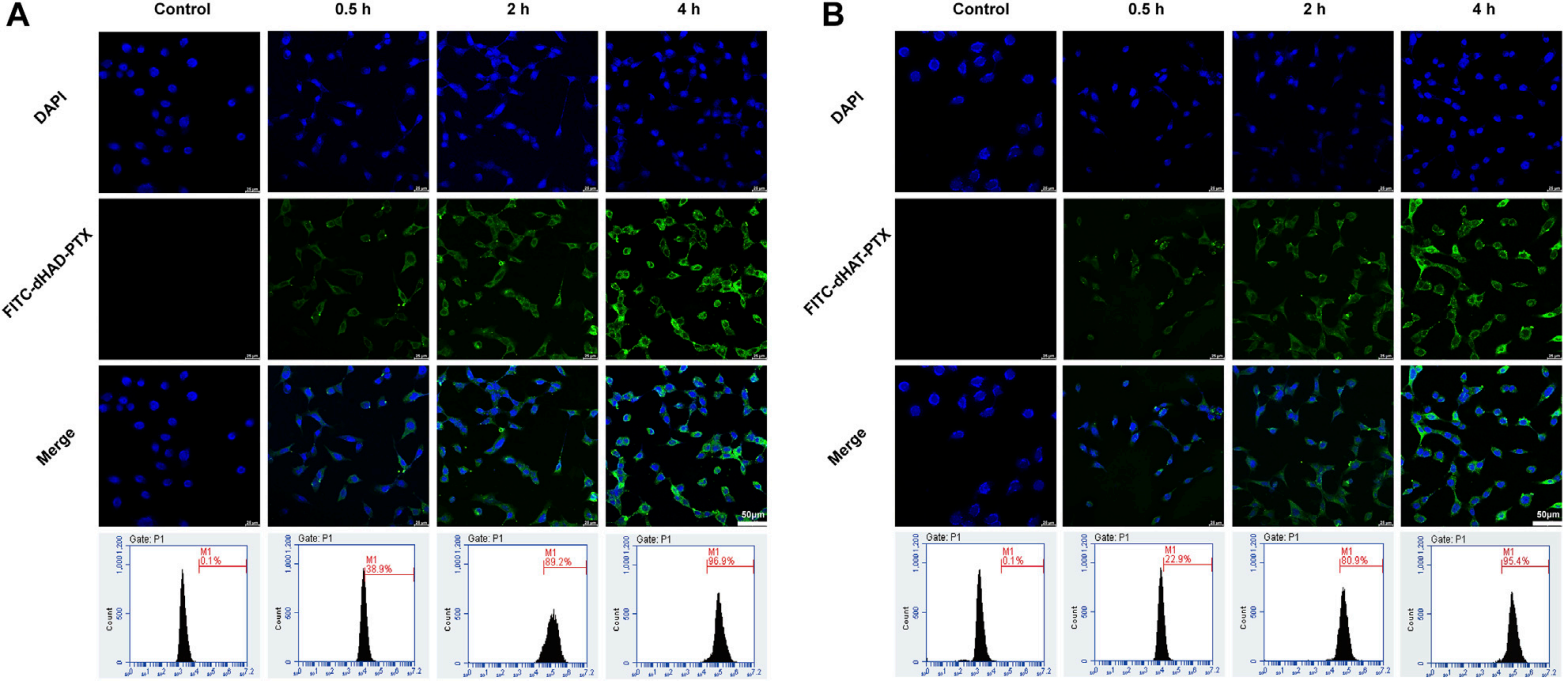
Analysis of *in vitro* cellular uptake of FITC-dHAD-PTX and FITC-dHAT-PTX micelles in MCF-7 cells. **(A, B)** CLSM and FCM analysis of MCF-7 cells treated with FITC-dHAD-PTX micelles **(A)** and FITC-dHAT-PTX micelles **(B)** for 0.5, 2, and 4 h. Scale bars: 50 μm.

Because CD44 is recognized by HA and is overexpressed by various tumors, HA-based carrier systems for tumor-targeted drug delivery are promising for precise chemotherapy. In this study, we qualitatively analyzed selective uptake of FITC-labeled dHAD-PTX and dHAT-PTX micelles by MCF-7 cells by using CLSM, and uptake was quantified by means of FCM. As shown in Figures 7A,B, after the cells were incubated with FITC-dHAD-PTX or FITC-dHAT-PTX micelles for 0.5, 2, and 4 h, the FITC mainly exists in cytoplasm, and fluorescence intensity increased with increasing incubation time. These results indicate that the drug-loaded micelles were gradually internalized by the cells via the CD44 receptors. Cellular uptake was confirmed and quantified by FCM. After 4 h of incubation, the uptake efficiency of MCF-7 cells for the dHAD-PTX micelles was 96.9% and that for the dHAT-PTX micelles was 95.4% (Figures 7A,B). That is, the MCF-7 cells efficiently internalized both types of micelles.

**FIGURE 8:**
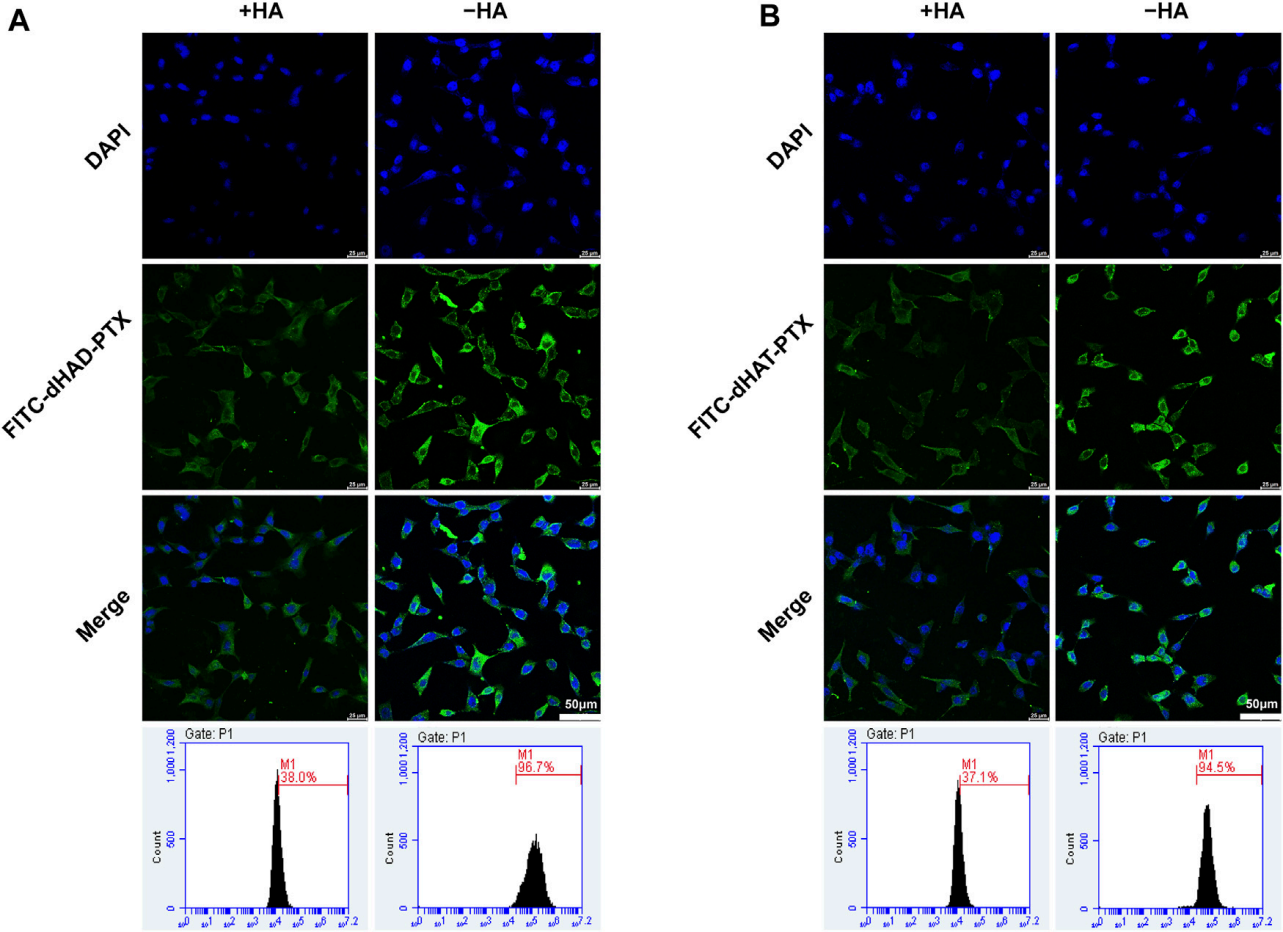
Analysis of *in vitro* targeting of FITC-dHAD-PTX and FITC-dHAT-PTX micelles in MCF-7 cells. **(A, B)** CLSM and FCM analysis of MCF-7 cells after culture with FITC-dHAD-PTX **(A)** and FITC-dHAT-PTX **(B)** for 4 h with or without pretreatment with free HA. Scale bars: 50 μm.

To verify the effect of HA on cell uptake, we preincubated MCF-7 cells with excessive HA to occupy cell surface CD44 receptors. Then the cells were incubated with FITC-dHAD-PTX or FITC-dHAT-PTX for 4 h and analyzed by CLSM and FCM (Figures 8A,B). These experiments showed that the intracellular fluorescence intensity was significantly lower than that observed for cells that had not been preincubated with HA, and the efficiencies of uptake of FITC-dHAD-PTX and FITC-dHAT-PTX micelles decreased to 38.0% and 37.1%, respectively. These results confirmed that preincubation with free HA interfered with binding of the drug-loaded micelles to CD44. Therefore, drug-loaded dHAD and dHAT micelles showed efficient HA-mediated cellular uptake. Chemotherapy drugs could be expected to be delivered to tumors through this special signaling pathway on the cell membrane.

### 3.8 *In vivo* biodistribution of the micelles

**FIGURE 9:**
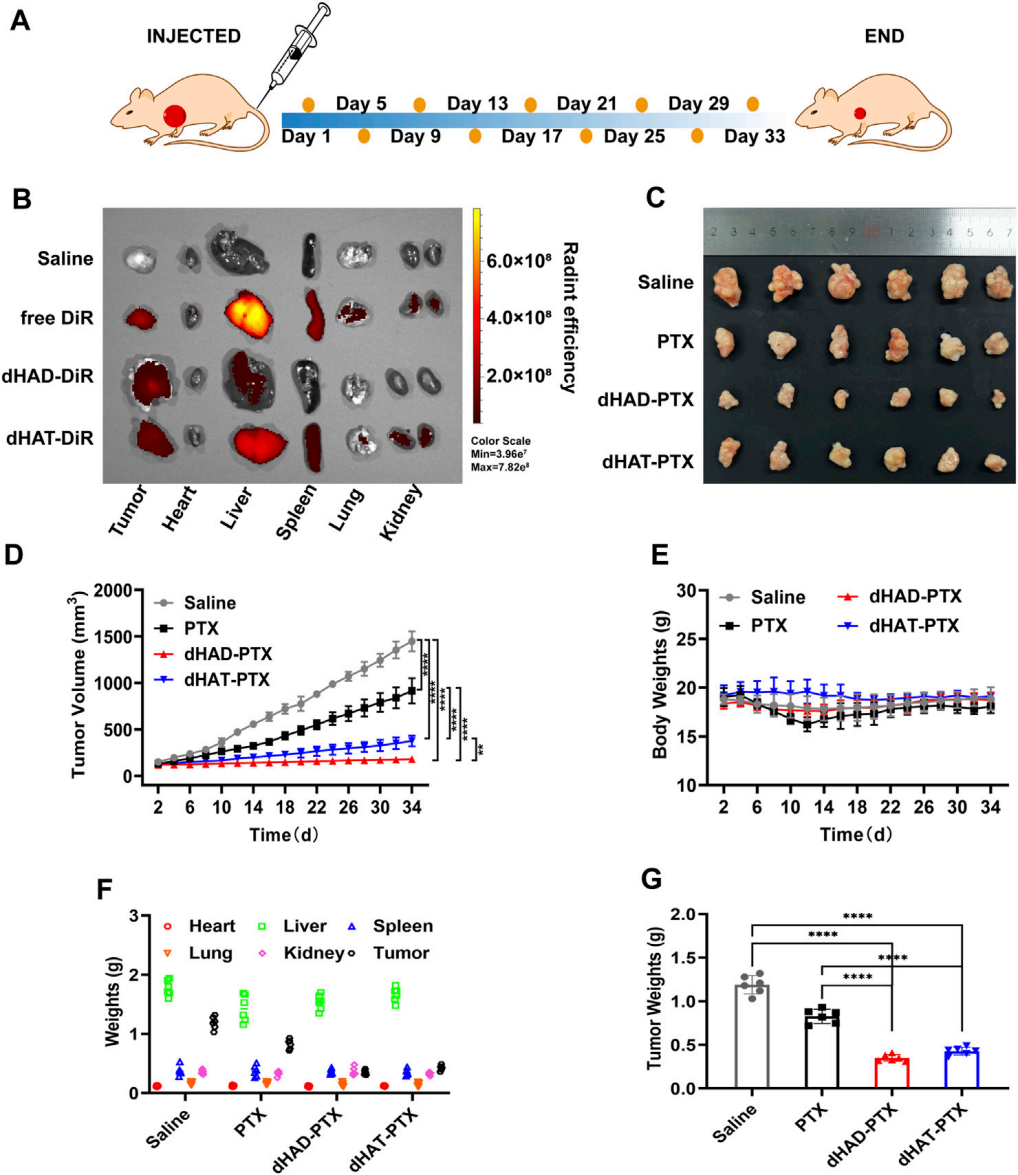
Comparison of the *in vivo* antitumor efficacies of drug-loading micelles, saline, and free PTX. **(A)** Treatment procedure. **(B)** *Ex vivo* fluorescence imaging of major organs and tumors of mice at 24 h after injection of saline, free DiR, dHAD-DiR, or dHAT-DiR. **(C)** *Ex vivo* images of tumors removed from MCF-7-tumor-bearing mice in different treatment groups. **(D)** Tumor growth curves (mean ± SD, n = 6; *p < 0.05, **p < 0.01, ***p < 0.001, ****p < 0.0001). **(E)** Changes of body weights of mice. **(F)** Weights of organs and tumors. **(G)** Tumor weight after 34 days of treatment (mean ± SD, n = 6; ****p < 0.0001).

To further evaluate the tumor targeting of micelles, we determined the tissue distribution and tumor accumulation of DiR, dHAD-DiR, and dHAT-DiR in MCF-7 tumor-bearing mice. Twenty-4 hours after injection, the tumors and major organs were removed, and the fluorescence distributions of DiR, dHAD-DiR and dHAT-DiR were determined. As shown in Figure 9B, some fluorescent signals could be observed in the livers, spleens, lungs and kidneys of the dHAT- DiR group. However, only a weak fluorescent signal was observed in the liver of the dHAD-DiR group. Free DiR fluorescence distribution could be observed throughout the body, but the fluorescence signal was stronger in the liver. As DiR could not form micelles, it showed systemic distribution and trace amount of tumor accumulation compared with DiR-loaded micelles. (Li et al., 2022). According to previous studies, nanoparticles or micelles with particle size distribution of about 100 nm can show strong accumulation at the tumor site (Supplementary Table S2) (Kibria et al., 2013; Liang et al., 2016).

### 3.9 *In vivo* antitumor effects of the micelles

As shown in Figure 9C and Supplementary Figure S9, the dHAD-PTX and dHAT-PTX micelles exhibited significantly better tumor suppressive efficacy *in vivo* than saline or free paclitaxel, and the dHAD-PTX micelles showed significantly better efficacy than the dHAT-PTX micelles. The tumor inhibition rates for the dHAD-PTX and dHAT-PTX micelles were 92.96% and 78.65%, respectively, whereas the value for free PTX was 58.46%, as determined from the tumor volumes (Figure 9D). The differences in tumor weights were consistent with the those in tumor volumes (Figure 9G).

During tumor inhibition, the dHAD-PTX and dHAT-PTX micelles increased PTX accumulation in the tumors by controlling its release, and these micelles inhibited tumor growth more efficiently than free PTX. The dHAD-PTX micelles responded rapidly to the low pH of the tumor microenvironment, which effectively improved their antitumor efficiency relative to free PTX (Supplementary Figure S8). Thus, the pH-responsive dHAD and dHAT micelles have great potential for antitumor applications.

### 3.10 *In vivo* evaluation of dHAD-PTX and dHAT-PTX micelle toxicity

**FIGURE 10:**
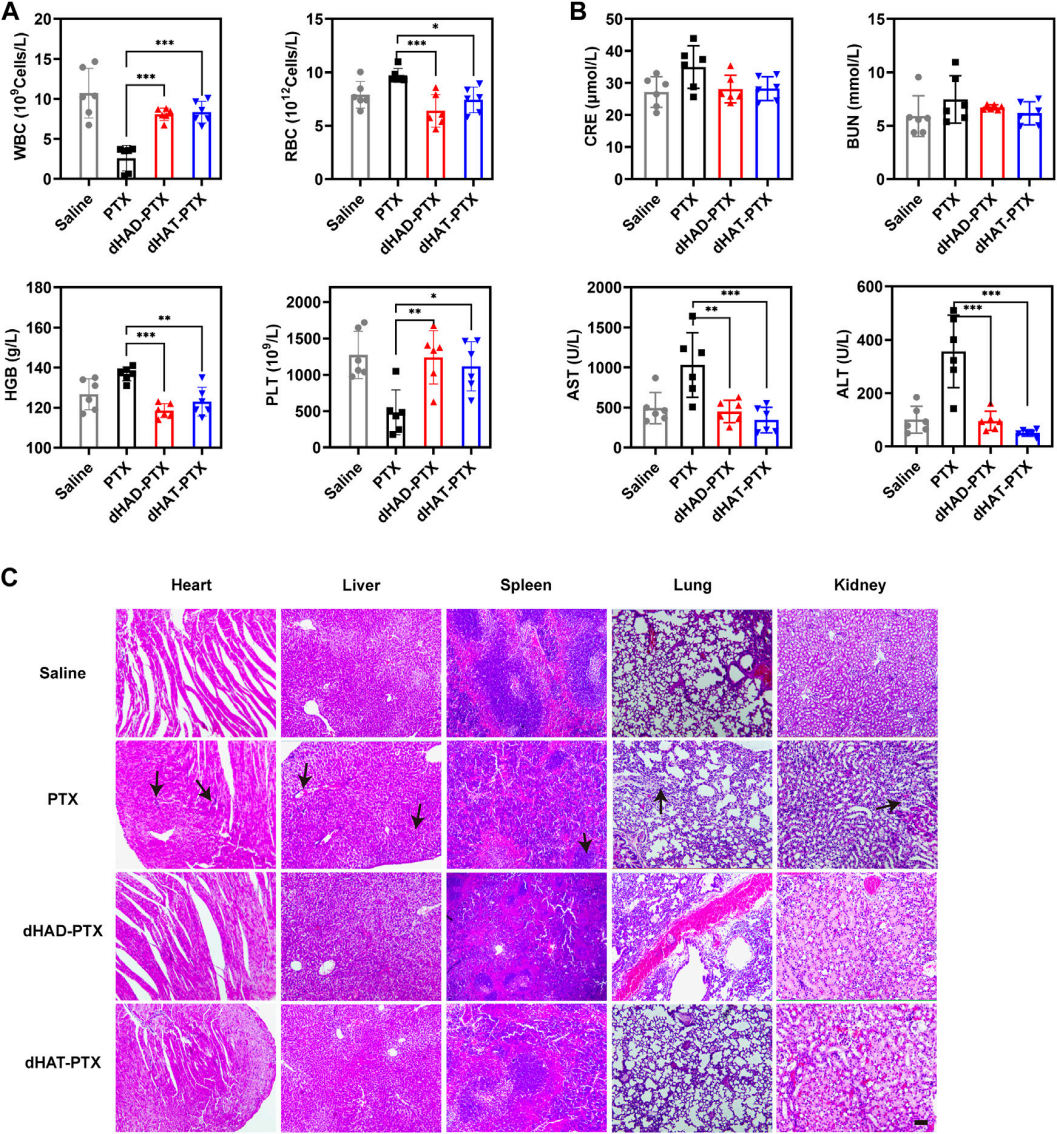
**(A)** Analysis of WBC and RBC counts, HGB levels, and PLT counts in blood. **(B)** Analysis of CRE, BUN, AST, and ALT levels in serum (mean ± SD, *n* = 6); *p < 0.05, **p < 0.01, ***p < 0.001). **(C)** H&E staining of heart, liver, spleen, lung, and kidney. Scale bar: 100 μm.

We assessed the *in vivo* toxicities of the dHAD-PTX and dHAT-PTX micelles on the basis of mouse body and organ weights, H&E staining, and hematological markers (Figures 10A,B). No significant changes in body or organ weights were observed in any of the groups (Figures 9E,F; Supplementary Figure S10). WBC counts, RBC counts, HGB levels, and PLT counts were used as hematological markers of toxicity. Serum ALT, AST, BUN, and CRE levels were used as markers of liver and kidney function, respectively; and their serum levels were used to assess liver and kidney toxicity. The measured values of these hematological markers for the dHAD-PTX- and dHAT-PTX-treated groups were comparable to those of the saline-treated group. PTX showed some hematological toxicity. There was little difference in liver or kidney function between the dHAD-PTX- and dHAT-PTX-treated mice and the saline-treated mice, whereas PTX showed potential hepatotoxicity and nephrotoxicity. The PTX group showed certain toxic side effects due to non-targeting. In contrast, dHAD-PTX and dHAT-PTX micelles showed good biocompatibility and almost no toxicity at experimental doses.

As shown in Figure 10C, H&E staining of the organs showed no obvious abnormalities or lesions in the dHAD-PTX- and dHAT-PTX-treated groups, whereas significant inflammation was observed in the PTX-treated group. In the PTX group, there was a large amount of inflammation in the hepatic lobule, and mild or moderate necrosis could be seen in the inflamed tissues. Partial hepatic sinusoid congestion was also observed. Inflammatory cell infiltration could be seen in most of the portal area. In the renal section, the glomeruli varied in size were extremely atrophic. We can also find moderate or severe proliferation of glomerular mesangial cells and mesangial matrix, tubulointerstitial infiltration of inflammatory cells and partial fibrosis. In summary, these results confirm that dHAD and dHAT polymers are safe carriers for chemotherapeutics.

## 4 Conclusion

In this study, we developed two pH-responsive amphiphilic HA-based drug carriers (designated as dHAD and dHAT), to enhance the efficacy of PTX for cancer therapy. The uptake efficiencies of dHAD-PTX and dHAT-PTX micelles into MCF-7 cells by means of CD44-mediated endocytosis were 96.9% and 95.4% at 4 h after treatment. Through active intratumoral targeting mediated by HA, the micelles indicated significant tumor inhibition both *in vitro* and *in vivo*; dHAD-PTX and dHAT-PTX could rapidly release drugs in the tumor microenvironment and remained stable under normal physiological conditions. The micelles with the same PTX equivalent dose had stronger inhibitory effects on MCF-7 cells than free PTX, and the *in vivo* tumor inhibition rates were 92.96% and 78.65%, respectively, which were higher than the 58.46% tumor suppression rate of free PTX. In addition, dHAD and dHAT displayed higher affinity for CD44 than HA did, the dHAD-PTX and dHAT-PTX micelles had good biocompatibility and negligible toxicity in mice. Taken together, our results indicate that dHAD-PTX and dHAT-PTX micelles with dual functions of tumor targeting and controlled drug release are promising drug carriers for antitumor therapy.

## Data Availability

The original contributions presented in the study are included in the article/Supplementary Material, further inquiries can be directed to the corresponding authors.
