# Supplementary material for: Dual functionalized hyaluronic acid micelles loading paclitaxel for the therapy of breast cancer
Source: Front Bioeng Biotechnol. 2023 Aug 3;11:1230585. doi: 10.3389/fbioe.2023.1230585 (PMC10436080; doi:10.3389/fbioe.2023.1230585)
Supplement: Supplementary file 2 [file DataSheet1.docx]

Supplementary Material

# Dual functionalized hyaluronic acid micelles loading paclitaxel for the therapy of breast cancer

Zhanbiao Liu^a,b^ †, Xuejun Chen^b^ †, Qian Jin^b^, Min Li^a^, Siqing Zhu^b^, Yi Zhang^b^, Defu Zhi^a^, Yinan Zhao^a,*^, Liqin Li^b,*^, Shubiao Zhang^a,*^

^a^*Key Laboratory of Biotechnology and* Bioresources Utilization of Ministry of Education, Dalian Minzu University, *Dalian 116600, PR China*.

^b^*State Key Laboratory of NBC Protection for Civilian, Beijing 102205, PR China.*

†These authors contributed equally to this work.

^*^Corresponding author.

Tel: (86) 0411-87505677

Fax: (86) 0411-87656215

E-mail: [zsb@dlnu.edu.cn](mailto:zsb@dlnu.edu.cn) (Shubiao Zhang)

**Supplementary Information**

***The preparation process of micelle loading FITC***

Weigh 1 mg of FITC, dissolve it in 100 μL of methanol, then add ultrapure water to 10 mL, and sonicate at 60 °C for 40 min to prepare a 200 μg/mL FITC solution. After diluting the prepared FITC solution to 100 μg/mL, 1 mg of drug-loaded micelles were dissolved in 1 mL of diluted FITC solution. After ultrasonication for 20 min, the FITC-labeled drug-loaded micelles were obtained by incubating on a shaker at 37 °C for 12 h.

**Supplementary Figures and Tables**

Fig. S1. Synthetic route of dHAT/D polymer.


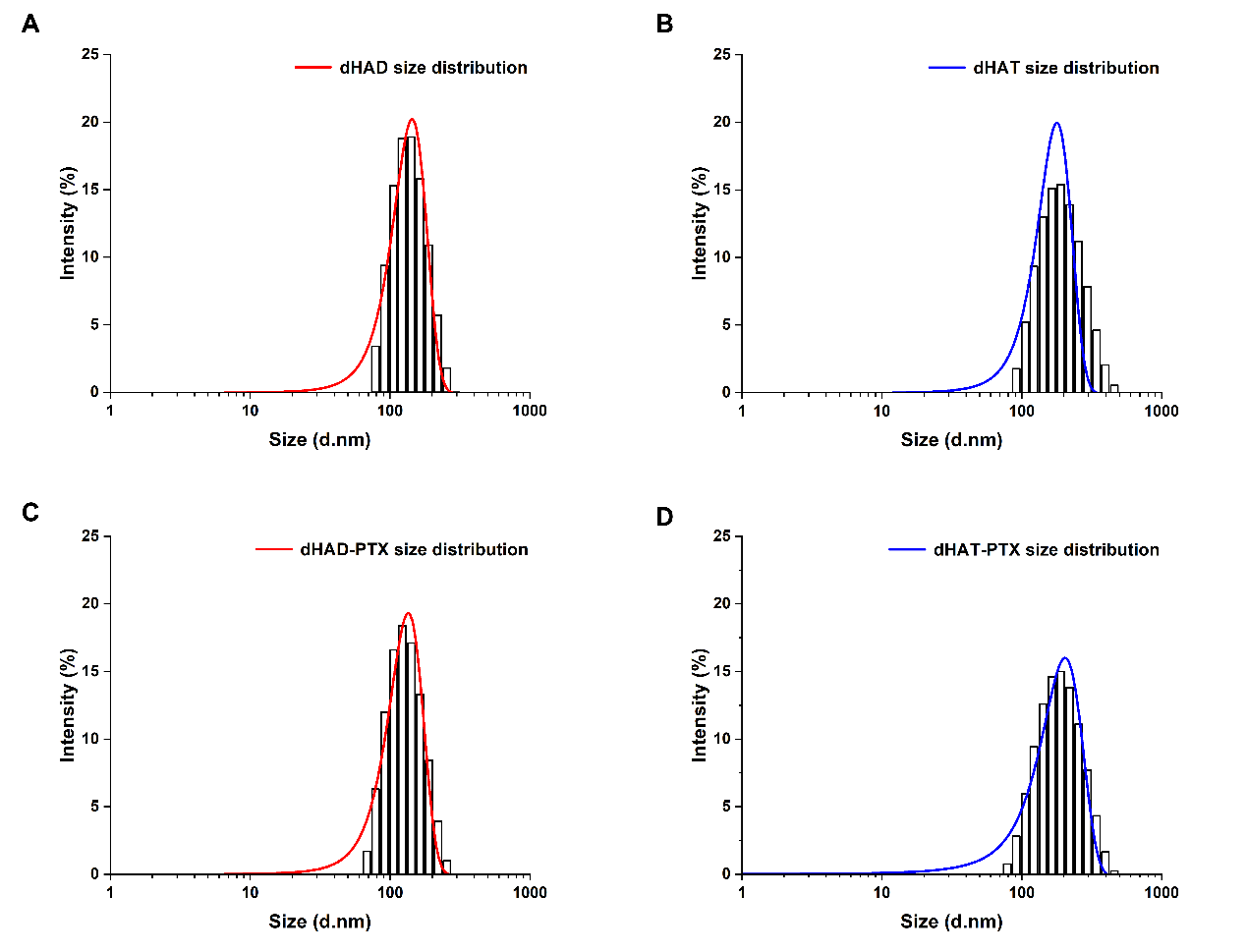


Fig. S2. Size distribution by DLS of dHAD micelles (A), dHAT micelles (B), dHAD-PTX micelles (C) and dHAT-PTX micelles (D).


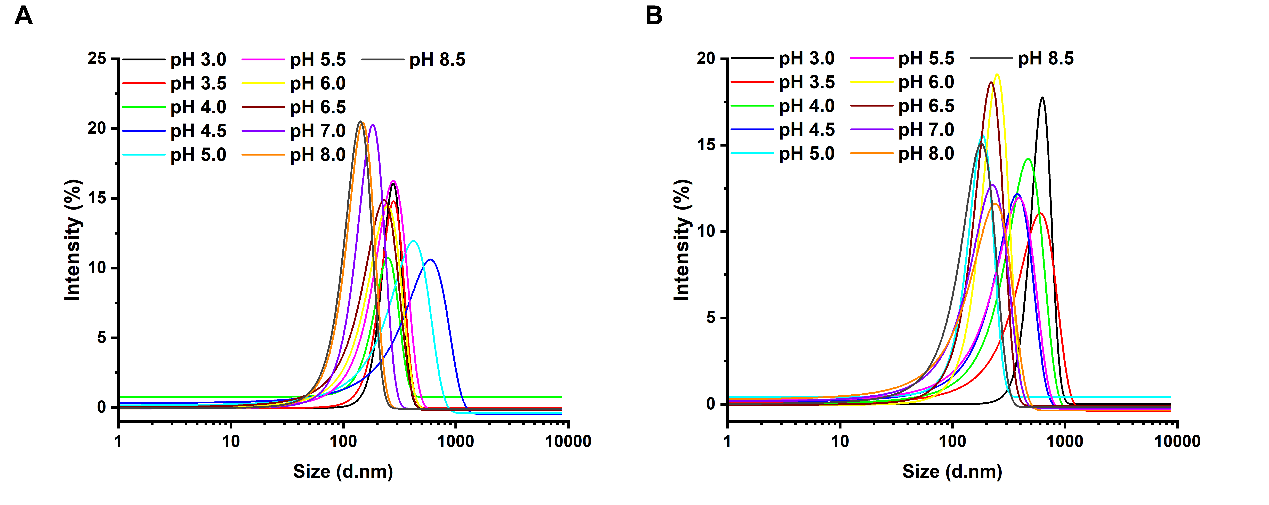


Fig. S3. Size distribution by DLS of dHAD-PTX (A) and dHAT-PTX (B) in pH 3.0-8.5.


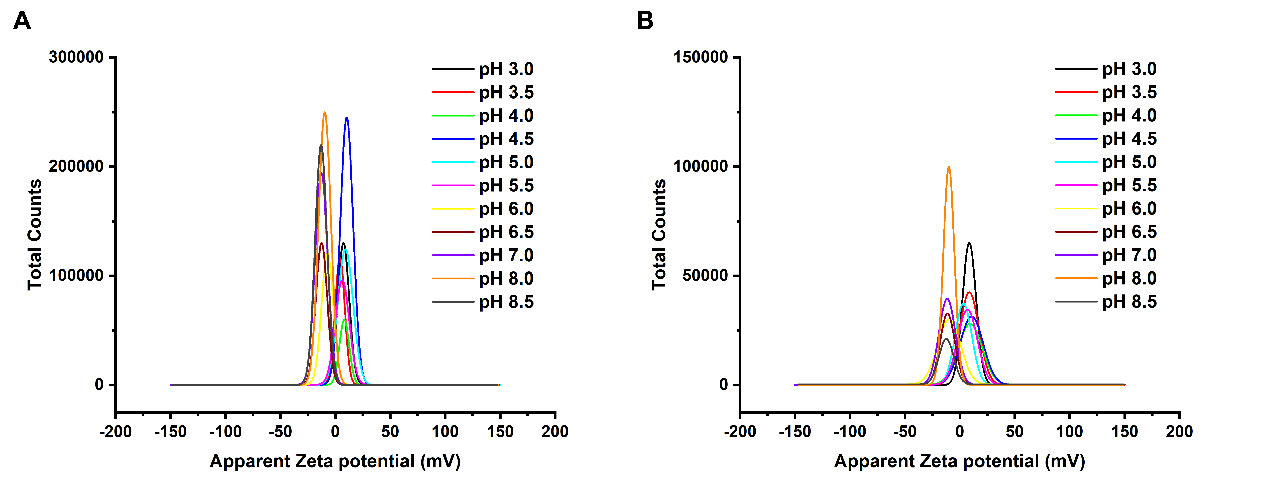


Fig. S4. Changes in zeta potential of dHAD-PTX (A) and dHAT-PTX (B) in pH 3.0-8.5.


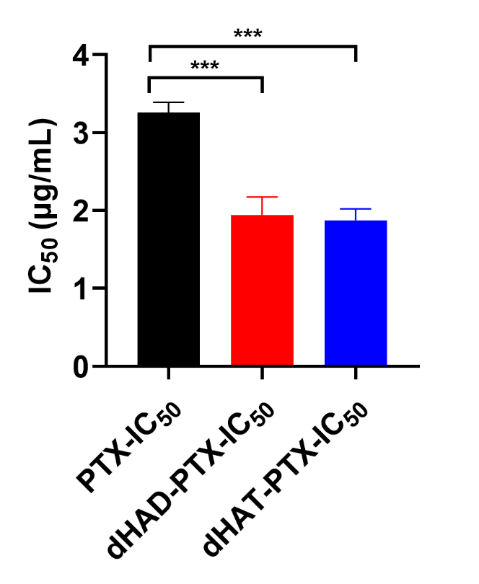


Fig. S5. Difference of IC_50_ in MCF-7 cells between dHAD-PTX, dHAT-PTX and free PTX (mean ± SD, n = 3); **p* < 0.05, ***p* < 0.01, ****p* < 0.001, *****p* < 0.0001).


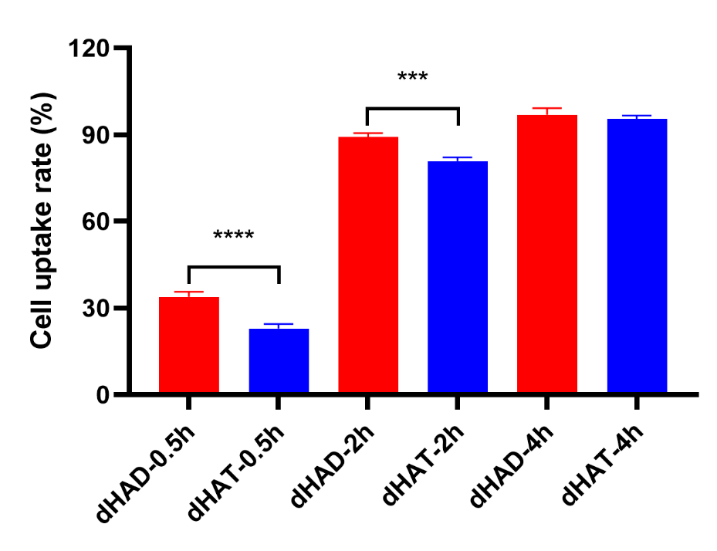


Fig. S6. Differences in cell uptake between dHAD and dHAT drug-loaded micelles (mean ± SD, n = 3); **p* < 0.05, ***p* < 0.01, ****p* < 0.001, *****p* < 0.0001).


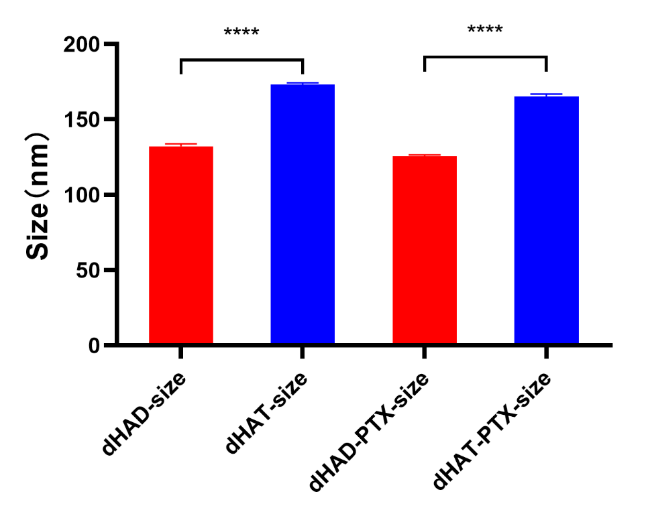


Fig. S7. Difference of particle size between polymers and drug-loaded micelles (mean ± SD, n = 5); **p* < 0.05, ***p* < 0.01, ****p* < 0.001, *****p* < 0.0001).


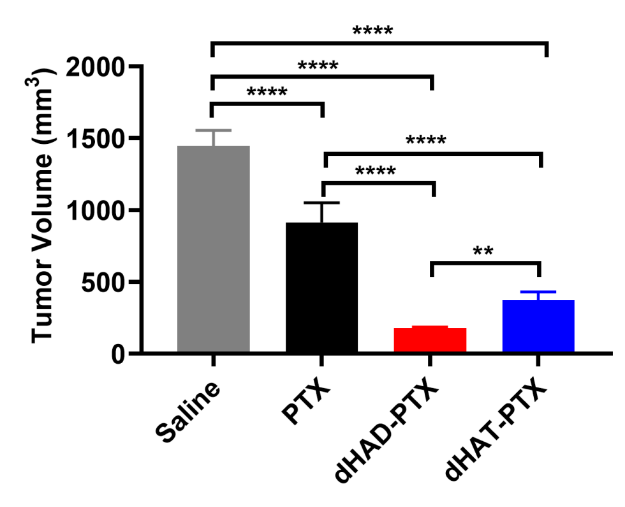


Fig. S8. The difference of tumor volume among different groups after treatment (mean ± SD, n = 6); **p* < 0.05, ***p* < 0.01, ****p* < 0.001, *****p* < 0.0001).


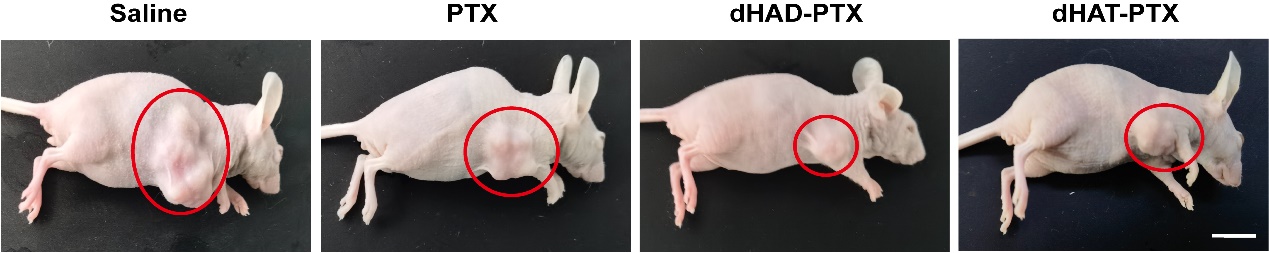


Fig. S9. Representative images of mice in different administration groups after treatment; one mouse was randomly selected from each group. The red circles indicate tumor extents. Scale bar: 1 cm.


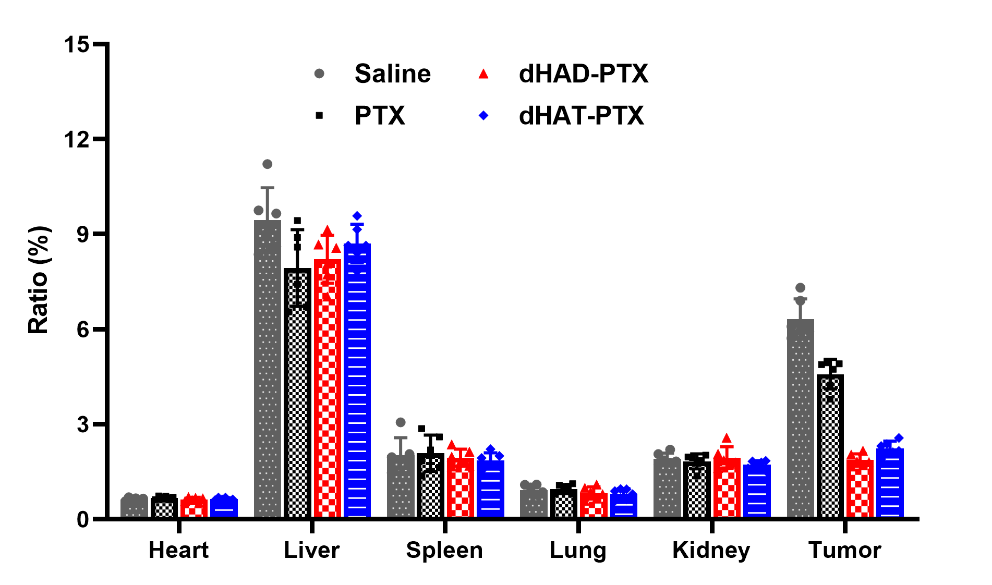


Fig. S10. The ratio of major organs to mouse body weight after 34 days of treatment (mean ± SD, n = 6).

Table S1. PDI distribution by DLS of dHAT/D-PTX micelles in pH 3.0-8.5 (mean ± SD, n = 3)

| Micelles | pH 3.0 | pH 3.5 | pH 4.0 | pH 4.5 | pH 5.0 | pH 5.5 | pH 6.0 | pH 6.5 | pH 7.5 | pH 8.0 | pH 8.5 |
| --- | --- | --- | --- | --- | --- | --- | --- | --- | --- | --- | --- |
| dHAD-PTX | 0.477±0.100 | 0.442±0.041 | 0.341±0.068 | 0.469±0.007 | 0.294±0.034 | 0.349±0.164 | 0.428±0.230 | 0.361±0.054 | 0.181±0.051 | 0.241±0.084 | 0.312±0.094 |
| dHAT-PTX | 0.599±0.143 | 0.470±0.085 | 0.525±0.080 | 0.585±0.138 | 0.445±0.041 | 0.586±0.105 | 0.481±0.140 | 0.536±0.044 | 0.201±0.086 | 0.311±0.025 | 0.412±0.157 |

Table S2. Properties of dHAD-DiR and dHAT-DiR micelles (mean ± SD, *n* = 5).

| Micelle | EE^a^ (%) | DL^b^ (%) | Size (nm) | PDI | Zeta (mV) |
| --- | --- | --- | --- | --- | --- |
| dHAD-DiR | 81.45 | 13.13 | 135.5±1.0 | 0.229±0.023 | −17.9±0.3 |
| dHAT-DiR | 81.12 | 13.25 | 175.3±1.5 | 0.195±0.085 | −16.8±0.2 |

Note: ^a^Encapsulation rate. ^b^Drug loading capacity.
